# ICEberg 3.0: functional categorization and analysis of the integrative and conjugative elements in bacteria

**DOI:** 10.1093/nar/gkad935

**Published:** 2023-10-23

**Authors:** Meng Wang, Guitian Liu, Meng Liu, Cui Tai, Zixin Deng, Jiangning Song, Hong-Yu Ou

**Affiliations:** State Key Laboratory of Microbial Metabolism, Joint International Laboratory on Metabolic & Developmental Sciences, School of Life Sciences & Biotechnology, Shanghai Jiao Tong University, Shanghai 200030, China; State Key Laboratory of Microbial Metabolism, Joint International Laboratory on Metabolic & Developmental Sciences, School of Life Sciences & Biotechnology, Shanghai Jiao Tong University, Shanghai 200030, China; State Key Laboratory of Microbial Metabolism, Joint International Laboratory on Metabolic & Developmental Sciences, School of Life Sciences & Biotechnology, Shanghai Jiao Tong University, Shanghai 200030, China; State Key Laboratory of Microbial Metabolism, Joint International Laboratory on Metabolic & Developmental Sciences, School of Life Sciences & Biotechnology, Shanghai Jiao Tong University, Shanghai 200030, China; State Key Laboratory of Microbial Metabolism, Joint International Laboratory on Metabolic & Developmental Sciences, School of Life Sciences & Biotechnology, Shanghai Jiao Tong University, Shanghai 200030, China; Biomedicine Discovery Institute and Department of Biochemistry and Molecular Biology, Monash University, Melbourne, VIC 3800, Australia; Monash Data Futures Institute, Monash University, Melbourne, VIC 3800, Australia; State Key Laboratory of Microbial Metabolism, Joint International Laboratory on Metabolic & Developmental Sciences, School of Life Sciences & Biotechnology, Shanghai Jiao Tong University, Shanghai 200030, China

## Abstract

**ICEberg 3.0** (https://tool2-mml.sjtu.edu.cn/ICEberg3/) is an upgraded database that provides comprehensive insights into bacterial integrative and conjugative elements (ICEs). In comparison to the previous version, three key enhancements were introduced: First, through text mining and manual curation, it now encompasses details of 2065 ICEs, 607 IMEs and 275 CIMEs, including 430 with experimental support. Secondly, ICEberg 3.0 systematically categorizes cargo gene functions of ICEs into six groups based on literature curation and predictive analysis, providing a profound understanding of ICEs’diverse biological traits. The cargo gene prediction pipeline is integrated into the online tool ICEfinder 2.0. Finally, ICEberg 3.0 aids the analysis and exploration of ICEs from the human microbiome. Extracted and manually curated from 2405 distinct human microbiome samples, the database comprises 1386 putative ICEs, offering insights into the complex dynamics of Bacteria-ICE-Cargo networks within the human microbiome. With the recent updates, ICEberg 3.0 enhances its capability to unravel the intricacies of ICE biology, particularly in the characterization and understanding of cargo gene functions and ICE interactions within the microbiome. This enhancement may facilitate the investigation of the dynamic landscape of ICE biology and its implications for microbial communities.

## Graphical Abstract

**Graphical Abstract:**
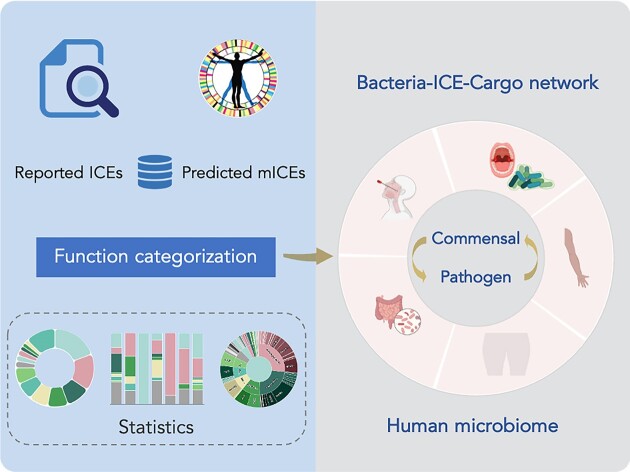

## Introduction

Integrative and conjugative elements (ICEs) are critical mobile genetic elements (MGEs) that play a significant role in bacterial evolution. They can integrate into the bacterial chromosome and possess entire conjugation machinery, thereby enabling self-transmission between bacterial cells (1). The modular nature of ICEs exerts a significant influence on shaping bacterial diversity and adaptation. They facilitate the horizontal transfer of diverse cargo genes, providing host bacteria with beneficial traits such as antibiotic resistance, pathogenesis, metal resistance, compound degradation and symbiosis, which contribute significantly to microbial evolution (2,3). Moreover, previously undisclosed cargo gene functions continue to emerge, for example, the *spbK* gene within the ICE*Bs1* element defends *Bacillus subtilis* against the SPß phage by utilizing the abortive infection system (4), and the BREX system within ICE*Vch*Ind5 shields *Vibrio cholerae* against phage attacks (5). Recently several studies have explored the distribution of diverse functions of ICEs (6,7); however, a systematic categorization framework of ICE cargo genes has not yet been conducted to date.

Over the past decade, growing research has focused on horizontal gene transfer within the human microbiome, especially concerning transfers linked to antibiotic resistance (8). Multiple metagenomic analyses of the human microbiome have indicated extensive horizontal gene transfer involving ICEs within the intricate and diverse microbial communities (9). The human microbiome serves as a significant and readily accessible reservoir of diverse functional genes (10). Such functional genes could be transferred between commensal bacteria or transferred to pathogens via MGEs, with ICEs standing out due to their proficiency in facilitating transfer across distant taxonomic groups (11,12). This capacity contributes to acquiring and disseminating beneficial traits among bacterial communities, thereby influencing the diversity of microbial populations. For instance, erythromycin resistance mediated by Tn*916*-like ICE has been implicated in conjugative transfer between viridans group streptococci (VGS) and other streptococci within the oral microbial community (13); intra- and inter-species transfer of ICE*St*3 has been observed under conditions simulating the human upper digestive tract (14). The wealth of genomic data from the human microbiome has paved the way for extensive exploration of ICEs within this domain.

In 2019, we reported the web-based open-access database ICEberg 2.0, archiving a dataset of curated ICEs along with an ICE/IME detection tool called ICEfinder 1.0 (15). As potent drivers of bacterial evolution, ICEs have garnered heightened attention, leading to the discovery of new ICEs with diverse functions after the ICEberg 2.0 release. Additionally, despite their growing importance, a comprehensive inventory of microbiome-associated ICEs is currently lacking, further highlighting the pressing need for an updated version. Here, we report the release of ICEberg version 3.0, reflecting a substantial expansion of the curated ICEs dataset, accompanied by a systematic categorization of functions. Notably, a new section has been introduced to archive and showcase ICEs and potential mobility networks of Bacteria-ICE-Cargo sourced from the human microbiome (Figure S1). We anticipate that ICEberg will provide enhanced support for researchers interested in bacterial horizontal gene transfer.

## Materials and methods

### Updating ICE data through text mining and manual curations

After manually curating the search results, we gathered 312 PubMed-archived papers published since 2019 into ICEberg 3.0, resulting in a compilation of 1006 papers in the database. We comprehensively extracted the essential information about ICEs from the literature, including element names, element families, host strains, recipients, insertion sites, boundaries, accession numbers, conjugative genes and accessory functional genes. The data accuracy and reliability were manually revisited (Table S1). Moreover, we reconstructed the website using the open-source framework AdminLTE, replacing outdated systems and introducing advanced features for an improved user experience. We utilized JavaScript and jQuery, integrating libraries like Svgene, D3, Highcharts, Echarts and AngularJS to enhance dynamic interaction and improve the visual representation of ICEs' modular structures and diverse cargo functions. In addition, the ICE prediction tool ICEfinder 2.0 was also updated to enhance the detection of ICE/IME in Gram-positive bacteria by integrating the CONJScan model from the macsyfinder software (16) and the partial relaxase data from ICEscreen (17) (Figure S2).

### Categorization of ICE cargo gene functions

As a crucial component and research hotspot of ICEs, cargo gene functions were systematically classified and integrated into the updated database (Figure S1). Initially, we established a comprehensive functional gene data set, incorporating antibiotic resistance genes sourced from Resfinder 4.1 (18), virulence factor genes obtained from VFDB (19), metal resistance genes from BacMet2 (20), microbial degradation genes from mibPOPdb (21) and symbiotic genes compiled from relevant literature. The identification of defense systems was executed using DefenseFinder v.1.0.9 (22). Subsequently, through a combination of literature curation and predictive analysis using BLASTp (*Ha*-value ≥ 0.64) (15), the cargo genes within ICEs were categorized into six groups: antibiotic resistance, virulence factor, metal resistance, defense system, degradation capabilities and symbiosis. Symbiosis here mainly refers to the nitrogen-fixing symbiosis of rhizobia. This refined categorization framework provides a profound comprehension of the biological traits and functionalities linked with ICEs. Additionally, the function gene dataset has been integrated into ICEfinder 2.0 (Figure S2), thereby facilitating researchers’ efforts to concurrently predict ICEs and obtain insights into their functional annotations.

### ICEs in human microbiome

A new section named ‘mICE Browse’ has been introduced, dedicated to archiving and showcasing putative ICEs predicted in the human microbiome (Table S2, S3). It's worth noting that ‘mICE’ stands for ICE derived from the microbiome. Initially, we extracted assembled sequences from 2405 samples of the Human Microbiome Project (HMP) in May 2023 (23,24), covering five body sites: the nasal cavity (266 samples), oral cavity (1296 samples), skin (57 samples), gastrointestinal tract (553 samples) and urogenital tract (233 samples). This compilation yielded a total of 144 257 778 assembled reads, with 2 456 785 reads longer than 10 kb being employed for subsequent analyses. Then, mICEs within the human microbiome were identified using the following process: the signature proteins of the conjugation module were detected utilizing CONJScan (16), and the integrative modules were identified based on profile HMMs as in our previous study (15). Moreover, the search for the *oriT* region was conducted using oriTfinder (25). To detect the 3′ termini of tRNA/tmRNA genes, indicative of potential insertion sites for mICEs, we utilized ARAGORN with the default parameters (26). Additionally, the Vmatch tool (http://vmatch.de/) was applied with the default configurations to recognize the directed repeats serving as the tRNA-distal boundaries. The mICE identify pipeline has been integrated into ICEfinder 2.0 (Figure S3).

Subsequently, the contigs containing mICE sequences were aligned with the PLSDB (27) plasmid database using Mash (28), and the identified matches (mash dist -v 0.1 -d 0.005) corresponding to plasmid elements were eliminated. For the taxonomic classification of mICE-containing contigs, Kraken2 was employed (29). Then a deduplication process was performed, removing identical mICEs originating from the same body site, host strain and possessing identical sequences. Finally, the retained mICEs were clustered using CD-Hit (-c 0.95 -n 10) (30). For each mICE cluster, we identified similar reported ICEs by CD-Hit. mICEs within the same cluster originating from different body sites and host strains were defined as potentially transferable mICEs. Leveraging these mICE clusters, we delineate the Bacteria-ICE-Cargo networks, providing a comprehensive view of their distribution and relationships.

## Results and discussion

Compared to the previous version, ICEberg 3.0 has been updated with the following three major improvements: (i) new ICE, IME and CIME data with manual curation; (ii) categorization of ICE cargo gene functions; (iii) prediction of ICEs from the human microbiome. Accordingly, ICEberg 3.0 may help in facilitating the understanding of bacterial evolution by capturing the diverse functions and characteristics of ICEs.

### A universal categorization framework for ICE cargo gene functions

Through a rigorous process of data collection, manual curation and systematic integration, we have substantially expanded the scope of ICEberg 3.0, enhancing its utility as a comprehensive mobile genetic elements database. Specifically, within ICEberg 3.0, we added 1033 newly identified ICEs, 347 IMEs, and 40 CIMEs. Among these additions, 123 elements have been experimentally validated. As a result, the compilation of genetic elements now encompasses 2065 ICEs, 607 IMEs and 275 CIMEs. Significantly, a substantial subset of 430 elements has undergone experimental validation, thereby underscoring the quality and precision of the database content (Table S1).

**Figure 1.**
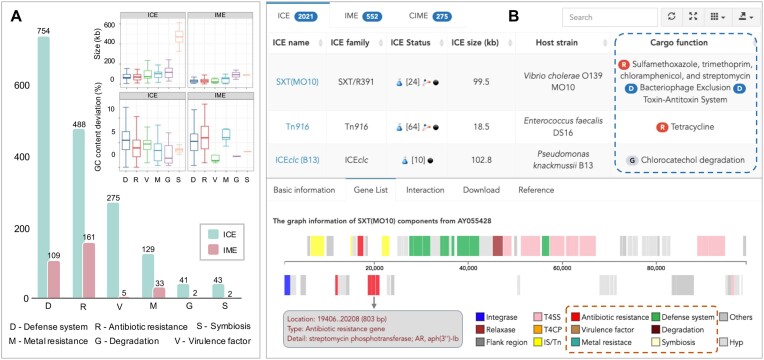
Overview of ICE cargo gene function categorization in ICEberg 3.0. (**A**) Statistical distribution of ICE/IME quantities across various functional categories within the database. The small graph in the upper right corner represents the size distribution and the GC content deviation (the absolute value of the GC content between ICE/IME and the host bacterial chromosome) of ICE/IMEs carrying different functions, with the X-axis matching the bar chart in the bottom left corner. (**B**) Tabular list and interactive visualization map displaying ICE detailed information on the ‘ICE Browse’ page.

The major focus of ICEberg 3.0 has been on the systematic classification and integration of cargo gene functions associated with ICEs. Through literature curation and predictive analysis, we effectively categorized prevalent cargo genes within ICEs into six distinct groups. These categories consist of 488 ICEs containing antibiotic resistance genes, 275 ICEs housing virulence factor genes, 754 ICEs carrying defense system genes, 129 ICEs with metal resistance genes, 41 ICEs featuring degradation genes and 43 ICEs harboring symbiotic genes, respectively. Furthermore, the ‘ICE Browse’ web page introduces categorized cargo gene function lists for individual ICEs, and the interactive presentations facilitate users to conveniently access detailed information about the genes within the function modules (Figure 1). This refined categorization framework provides a deeper understanding of the multifaceted biological traits and functionalities intrinsic to ICEs. Furthermore, we integrated the functional gene dataset into the backend of the ICE prediction tool ICEfinder 2.0 (Figure S2 and Table S4), facilitating the concurrent prediction of ICEs and providing researchers with insights into their functional annotations, offering further insights into the captivating realm of ICE biology.

### Bacteria-ICE-Cargo network in human microbiome

**Figure 2.**
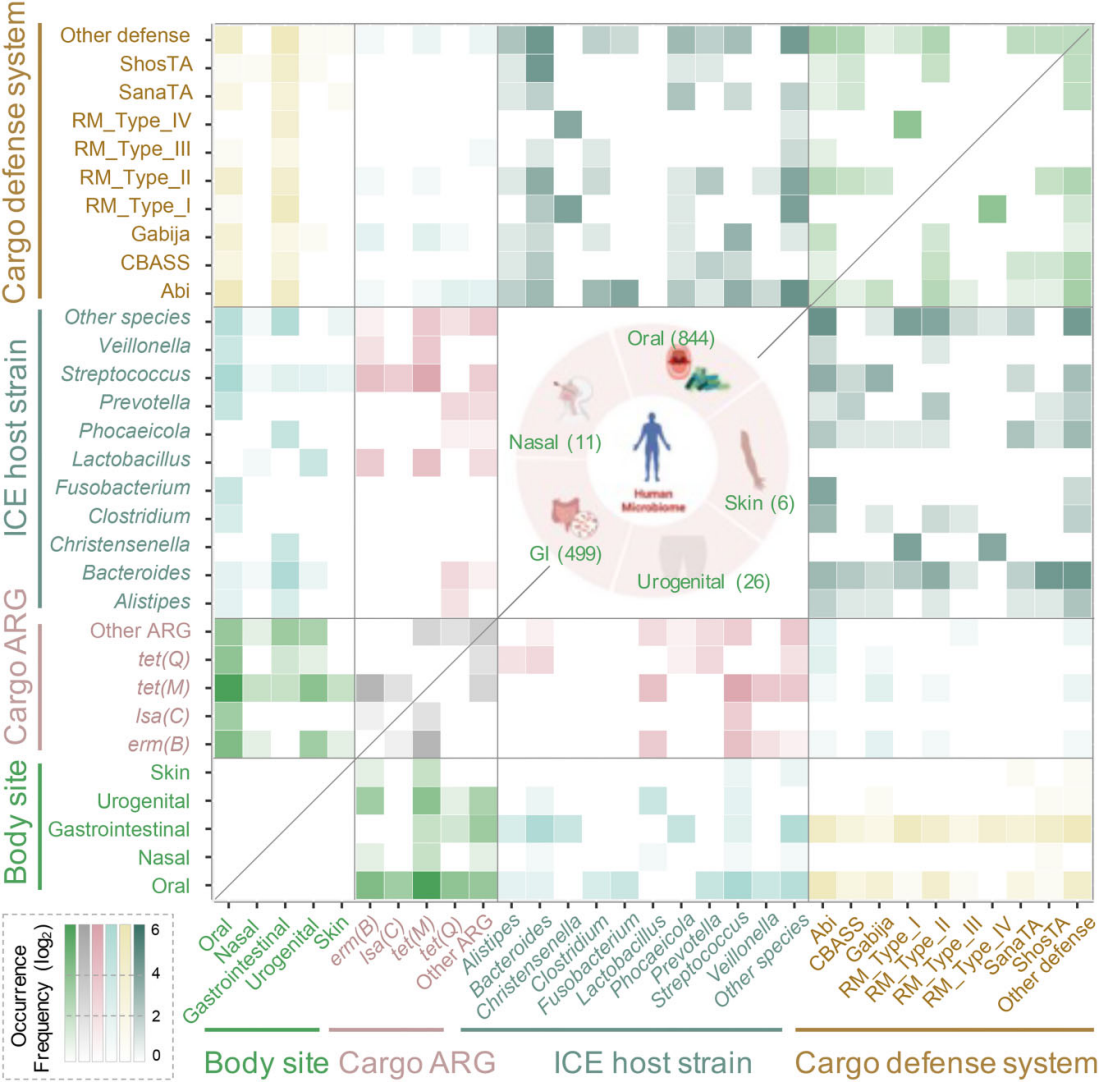
The relationship network between human body sites, ICE host strains, ICE cargo antibiotic resistance genes (ARGs) and ICE cargo defense systems cross ICEs within the human microbiome. All statistical data are derived from metagenomic ICEs associated with various body sites, represented in the central part of the figure. The numerical values indicate the respective quantities of ICEs derived from the microbiome of the corresponding body sites. Abbreviation: GI, gastrointestinal.

Based on the assembled sequences of 2405 samples acquired from the HMP, we have gathered a total of 1386 mICEs from the human microbiome. This collection includes 11 mICEs originating from the nasal cavity, 844 from the oral cavity, 6 from the skin, 499 from the gastrointestinal tract and 26 from the urogenital tract, respectively (Tables S2 and S3). The greater abundance of mICEs in the gastrointestinal and oral tissues can be attributed to the increased availability of research data about these areas. We have developed an extensive archive and visualization platform dedicated to these mICEs within the human microbiome, hosted on the ‘mICE Browse’ web page. This dataset of mICEs stands poised to evolve into a valuable reference resource, offering significant value to researchers engaged in the study of MGEs within the human microbiome, particularly concerning their associations with antibiotic resistance and defense systems. Analysis of the mICE dataset revealed the role of human microbiome ICEs as a crucial reservoir of antibiotic resistance genes (Figure 2), prominently featuring tetracycline resistance genes *tet(M)* and *tet(Q)*, in addition to the MLS resistance gene *erm(B)*. These resistance genes are predominantly distributed in the oral and urogenital tracts, enriched in *Streptococcus* and *Lactobacillus* species, with *tet(M)* and *erm(B)* often coexisting within the same ICE. Notably, aside from antibiotic resistance genes, we also identified a significant presence of defense systems carried by ICEs in the human microbiome, with the main systems of Restriction-Modification (RM) and Abortive Infection (Abi). These defense systems are primarily distributed in the oral and gastrointestinal tracts, with the RM systems enriched in *Bacteroides* and *Christensenella* species, while the Abi systems are mainly found in *Streptococcus* and *Fusobacterium*. These cargo genes may undergo horizontal transfer via ICEs between different symbiotic bacteria, potentially even to potential pathogens, enhancing their survival capabilities in complex environments. Within the mICE clusters, those carrying the tetracycline resistance gene *tet(M)* and affiliated with the Tn*916* family hold the most pronounced presence. This presence spans various bacterial species and different body sites, suggesting the crucial role of the Tn*916* family in the horizontal transfer of Antibiotic Resistance Genes (ARGs) within the human microbiome (Figures S4 and S5). Overall, the introduction of the expansive mICE dataset is helpful for our comprehension of the intricate interplay and dynamics within the Bacteria-ICE-Cargo network. This dataset empowers researchers to unravel the multifaceted roles that ICEs may undertake within the complex milieu of the human microbiota.

## Conclusion

We report a major update of ICEberg, a public database focusing on the comprehensive characterization of ICEs and their associated cargo functions. In addition, we also introduce a novel dataset of predicted ICEs sourced from the human microbiome. Furthermore, the integration of cargo gene function prediction and mICE identification into ICEfinder 2.0 enhances its capacity to delve into the multifaceted roles of ICEs within microbial communities. This enhanced ICE-specific resource was designed to assist researchers in exploring bacterial evolution, mobile genetic elements and ICE interactions with the human microbiome. The ICEberg database will be continuously maintained and updated to keep up with the rapidly expanding microbial genome database and ensure its ongoing relevance.

## Supplementary Material

gkad935_Supplemental_Files

## Data Availability

ICEberg3 is freely available at https://tool2-mml.sjtu.edu.cn/ICEberg3/.
