# Supplementary material for: ICEberg 3.0: functional categorization and analysis of the integrative and conjugative elements in bacteria
Source: Nucleic Acids Res. 2023 Oct 23;52(D1):D732–7. doi: 10.1093/nar/gkad935 (PMC10767825; doi:10.1093/nar/gkad935)
Supplement: gkad935_Supplemental_Files [file gkad935_supplemental_files.zip › Supplementary_file_HYO2.pdf]

ICEberg 3.0: functional categorization and analysis of the integrative and conjugative elements in bacteria

SUPPLEMENTARY DATA

Table S1. Statistical summary of T4SS-type ICEs, AICEs, IMEs and CIMEs archived in ICEberg 3.0.

Table S2. Statistical summary of the microbiome sequences in different human body sites sourced from the Human Microbiome Project (HMP) in May 2023.

Table S3. Detailed information on putative ICEs identified from the Human Microbiome Project (HMP).

Table S4. Comparison of ICEscreen, CONJScan and ICEfinder 2.0 for the prediction of 31 reference ICE/IMEs with experimental support.

Figure S1. Workflow of ICEberg 3.0 database construction.

Figure S2. The prediction strategy used by ICEfinder 2.0.

Figure S3. An overview of ICEfinder outputs using an example of the assembled contigs from the Human Microbiome Project (HMP).

Figure S4. An overview of the 1,386 mICEs identified from the Human Microbiome Project (HMP).

Figure S5. Example of visualization of the mICE cluster C1 from the ICEberg website.

Table S1. Statistical summary of T4SS-type ICEs, AICEs, IMEs and CIMEs archived in ICEberg 3.0.

	**Number of elements derived from experimental data**	**Number of elements predicted from the literature**	**Total**
ICEberg 1.0 (released in 2012)			
T4SS-type ICE	181	220	401
AICE	5	22	27
ICEberg 2.0 (released in 2019)			
T4SS-type ICE	259	592	851
AICE	11	170	181
IME	32	228	260
CIME	5	230	235
ICEberg 3.0 (updated in 2023)			
T4SS-type ICE	376	1,473	1,849
AICE	12	204	216
IME	37	570	607
CIME	5	270	275
mICE	-	1,386[a]	1,386

[a] mICEs were identified from the microbiome sequences of the Human Microbiome Project (Table S2).

Table S2. Statistical summary of the microbiome sequences in different human body sites, sourced from the Human Microbiome Project (HMP) in May 2023.

Body site	Sample count	Reads count (>10kb)	Reads mean length (kb)	mICE count
Nasal	266	17,123	28,476	11
Oral	1,296	1,217,220	19,551	844
Skin	57	21,047	27,041	6
Gastrointestinal	553	1,176,509	22,032	499
Urogenital	233	24,885	27,826	26
Total	2,405	2,456,784	24,985	1,386

Table S4. Comparison of ICEscreen, CONJScan and ICEfinder 2.0 for the prediction of 31 reference ICE/IMEs with experimental support.

Gram type	Genome Strain (NCBI accession no.)	Reference [a]		ICEscreen		CONJScan		ICEfinder 2.0				
		Element name & position	Size (bp)	Region detected	Size (bp)	Region detected	Size (bp)	Region detected	Size (bp)	Boundaries [b]	*oriT* [b]	function [c]
Gram+	*Bacillus subtilis* subsp. subtilis str. 168 (NC_000964.3)	ICE*Bs*1: 529362-549932	20570	529505-541229	11724	532922-545011	12089	529362-549932	20570	Y	Y	DS
Gram+	*Lactococcus lactis* subsp. lactis KF147 (NC_013656.1)	Tn*6098*: 2295682-2347036	51354	2295829-2310916	15087	2301659-2311432	9773	2295828-2308916	13088	-	-	-
Gram+	*Streptococcus agalactiae* strain GBS1-NY (NZ_CP007570)	ICE vanG-1: 605437-651025	45588	626101-651329	25228	626101-683362	57261	626100-695741	69641	-	-	DS
		ICE-r: 651023-695707	44684	653227-695741	42514							
Gram+	*Streptococcus agalactiae* strain GBS2-NM (NZ_CP007571)	ICE vanG-2: 641999-691323	49324	667177-691627	24450	667177-723650	56473	667176-736029	68853	-	-	DS
		ICE-r: 691321-735995	44674	693525-736029	42504							
Gram+	*Streptococcus anginosus* strain NCTC11064 (NZ_LR594037)	IME_*San*NCTC11064_oriT: 1499118-1500447	1329	1493940-1512815	18875	1471542-1559690	88148	1498128-1512815	14687	-	Y	-
Gram+	*Streptococcus pyogenes* MGAS6180 (NC_007296.2)	ICE 6180-RD.2: 1286381-1322707	36326	1286472-1303895	17423	1294399-1303895	9496	1286325-1301874	15549	-	-	-
Gram+	*Streptococcus* sp. FDAARGOS_522 (NZ_CP033808)	IME: 932328-937510	5182	935192-937510	2318	929902-946833	16931	929901-952945	23044	-	Y	AR
Gram+	*Streptococcus suis* D9 (NC_017620.1)	ICE_*Ssu*D9_rplL: 1033490-1089187	55697	1033550-1084280	50730	1035684-1146013	110329	1017839-1146013	128174	-	-	AR
Gram+	*Streptococcus suis* strain HN105 (NZ_CP029398)	ICE*SSu*HN105: 951385-1031024	79639	960912-1030966	70054	954075-1017858	63783	954074-1046239	92165	-	-	DS; AR
Gram+	*Bacillus subtilis* subsp. subtilis str. 168 (AL009126)	ICE*Bs*1: 529423-549932	20509	529505-541229	11724	532922-545011	12089	529362-549932	20570	Y	Y	DS
Gram+	*Streptococcus mutans* UA159 (AE014133)	Tn*Smu*1: 192029-206864	14835	192029-206423	14394	197498-206864	9366	191935-212500	20565	Y	-	-
Gram+	*Saccharopolyspora erythraea* NRRL 2338 (AM420293)	pSE211 [AICE]: 7823295-7840549	17254	-	-	-	-	7823295-7840549	17254	Y	-	-
Gram+	*Streptomyces coelicolor* A3(2) (NC_003888)	SLP1 [AICE]: 5038594-5055797	17203	-	-	-	-	5038539-5055705	17166	Y	-	-
Gram-	*Salmonella enterica* subsp. enterica serovar Senftenberg, 5494-57 (FN298496)	CTnscr94: 11966-126312	114346	47630-61302	13672	26814-124991	98177	11964-126362	114398	Y	-	DS

Gram-	*Escherichia coli* UMN026 (CU928163)	ICE*Eco*UMN026-1: 2277431-2343162	65731	2313230-2327766	14536	2312214-2327766	15552	2277415-2343163	65748	Y	Y	DS; VF
Gram-	*Glaesserella parasuis* EHP1804 (CP069308.1)	ICE*Gpa*1804: 717521-789400	71879	736496-747374	10878	733775-788335	54560	733775-789229	55454	-	-	AR
Gram-	*H. parasuis* YHP170504 (CP054198.1)	ICE*Hpa*1: 1511383-1580304	68921	1551874-1562752	10878	1512492-1565473	52981	1511598-1526231	14633	-	-	AR
Gram-	*Klebsiella pneumoniae* NTUH-K2044 (AP006725)	ICE*Kp*1: 3395836-3471770	75934	3450241-3463548	13307	3449225-3463548	14323	3395820-3472027	76207	Y	Y	VF
Gram-	*Mesorhizobium ciceri* CC1192 (CP015062.1)	ICE*Mc*Sym1192: 4216411-4635338	418927	4242077-4268112	26035	4237029-4268112	31083	4216411-4635339	418928	Y	-	DS; VF
Gram-	*Proteus mirabilis* HI4320 (AM942759)	ICE*Pm*1: 2793762-2886224	92462	2793999-2826218	32219	2795085-2871627	76542	2793715-2886352	92637	Y	-	VF
Gram-	*Salmonella bongori* CEIM46082 (FN298494)	ICE*Sb*1: 1785-111202	109417	37989-111024	73035	16075-109847	93772	1784-111253	109469	Y	-	VF
Gram-	*Y. hibernica* CFS1934 (CP032487.1)	ICE*Yh*1: 3787208-3893449	106241	3787515-3863185	75670	3768997-3874457	105460	3787208-3893449	106241	Y	-	DS; VF
Gram-	*Legionella pneumophila* Corby (CP000675.2)	LpcGI-2: 2781725-2846914	65189	2781725-2826507	44782	2809766-2826507	16741	2809766-2826507	16741	-	-	-
Gram-	*Pseudomonas aeruginosa* C (AF440523)	PAGI-2: 27286-132240	104954	73749-91885	18136	63484-94997	31513	63484-94997	31513	-	-	DS
Gram-	*Pseudomonas aeruginosa* UCBPP-PA14 (CP000438)	PAPI-1: 5251440-5359392	107952	5310109-5339996	29887	5234279-5357743	123464	5251438-5359437	107999	Y	-	DS; VF
Gram-	*Pseudomonas syringae* pv. phaseolicola 1302A (AJ870974.1)	PPHGI-1: 1298-107263	105965	75872-106805	30933	18737-105387	86650	1298-107263	105965	Y	-	DS; VF
Gram-	*Enterobacter* sp. A1137 (CP021851.1)	Tn*6397*: 386444-510401	123957	403705-454282	50577	399346-422656	23310	386441-510401	123960	Y	-	DS; AR; MR
Gram-	*Legionella pneumophila* Corby (CP000675)	Trb-1: 614497-656813	42316	620993-637175	16182	619346-637175	17829	615234-637175	21941		-	VF
Gram-	*Yersinia pseudotuberculosis* 32777 (AJ627388)	YAPI: 1402-99513	98111	25685-99213	73528	11007-98203	87196	1402-99513	98111	Y	-	DS; VF

[a] The reference ICE position was taken from the literature and recorded by the database ICEberg 3.0. The position mentioned in the articles was calculated based on the boundary genes sometimes, which might result in slight discrepancies with the DR positions we predicted by ICEfinder 2.0.

[b] Y indicates DR boundaries or *oriT* sequences were detected.

[c] DS: Defense system; AR: Antibiotic resistance; VF: Virulence factor; MR: Metal resistance.

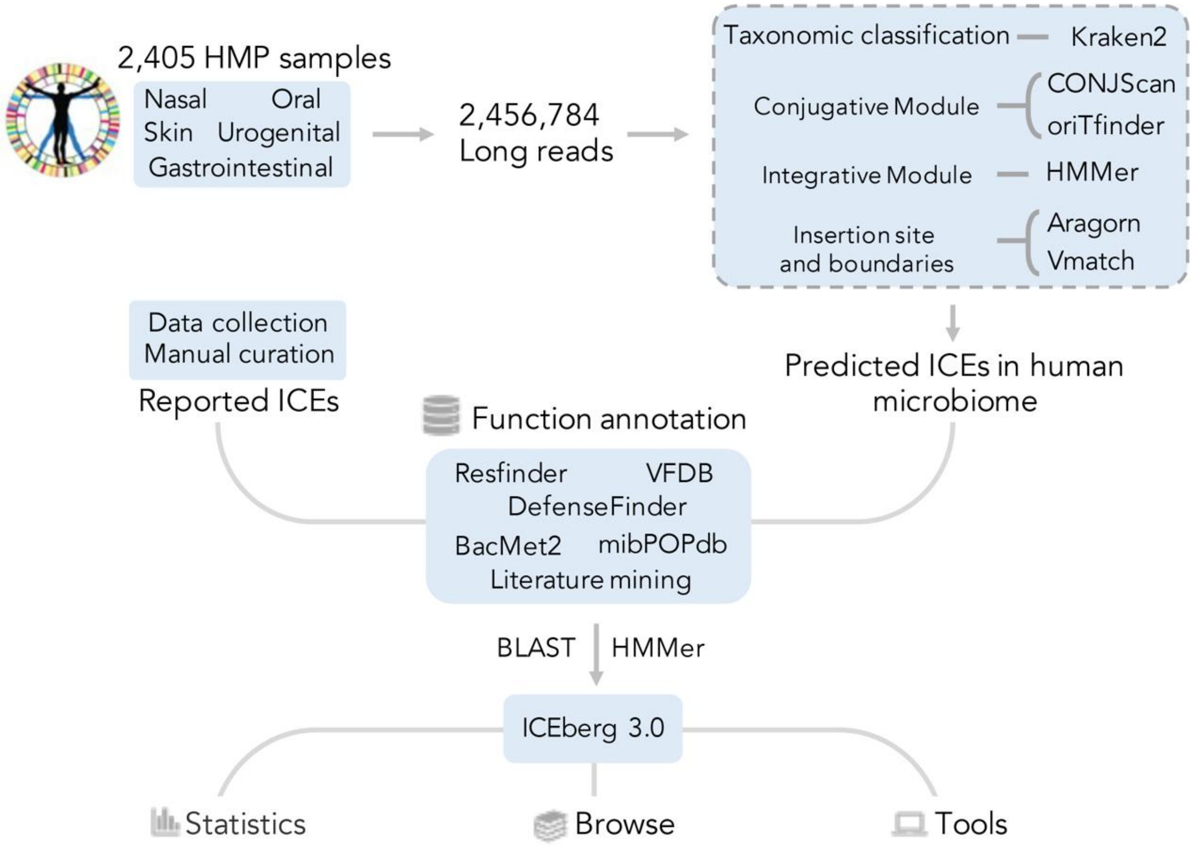

Figure S1. Workflow of ICEberg 3.0 database construction.

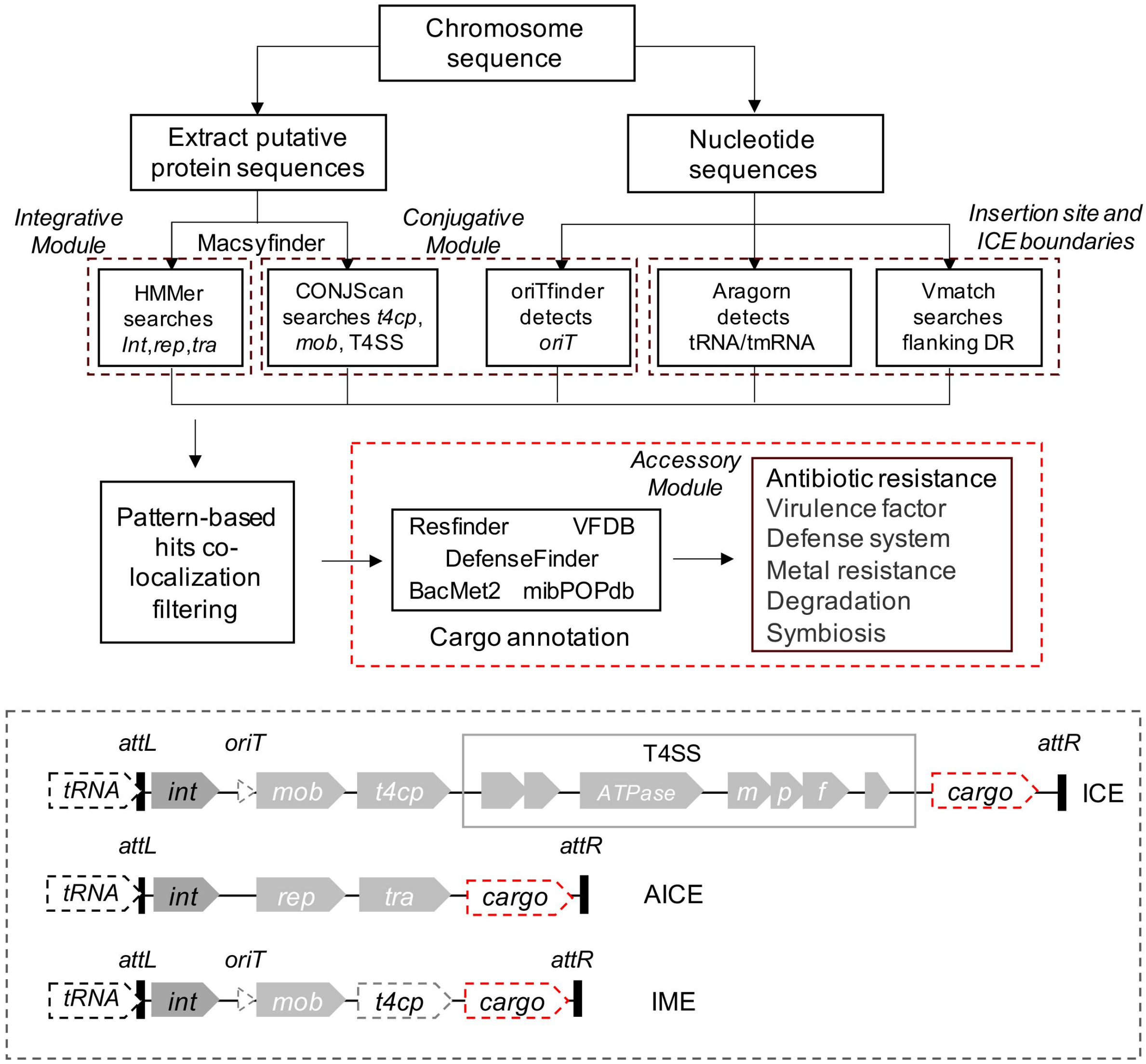

Figure S2. The prediction strategy used by ICEfinder 2.0. The integrative module, conjugative module, insertion site, and boundaries of ICE were individually identified. To enhance the detection of ICE/IME in Gram-positive bacteria, ICEfinder 2.0 integrated the CONJScan model from the macsyfinder software and the partial relaxase data from ICEscreen. To locate the 3′ end of tRNA/tmRNA genes, which indicate potential insertion sites, ICEfinder 2.0 employed ARAGORN. ICEfinder 2.0 utilized the Vmatch tool to recognize directed repeats that serve as the tRNA gene-distal boundaries. ICEfinder 2.0 annotated the cargo genes using various databases and tools. A comparative analysis of three ICE prediction tools (ICEscreen, CONJScan and ICEfinder 2.0) using 31 experimentally validated ICE/IME samples from various bacterial species, including 13 Firmicutes, 2 Actinobacteria, and 16 Gram-negative bacteria, is shown in Table S4. ICEfinder 2.0 gave a comparable prediction accuracy for these reference ICEs with ICEscreen and CONJScan. Notably, ICEfinder was capable of predicting AICE in actinomycetes. In addition, for the ICEs inserted at tRNA/tmRNA genes, ICEfinder exhibited precise DR boundary predictions.

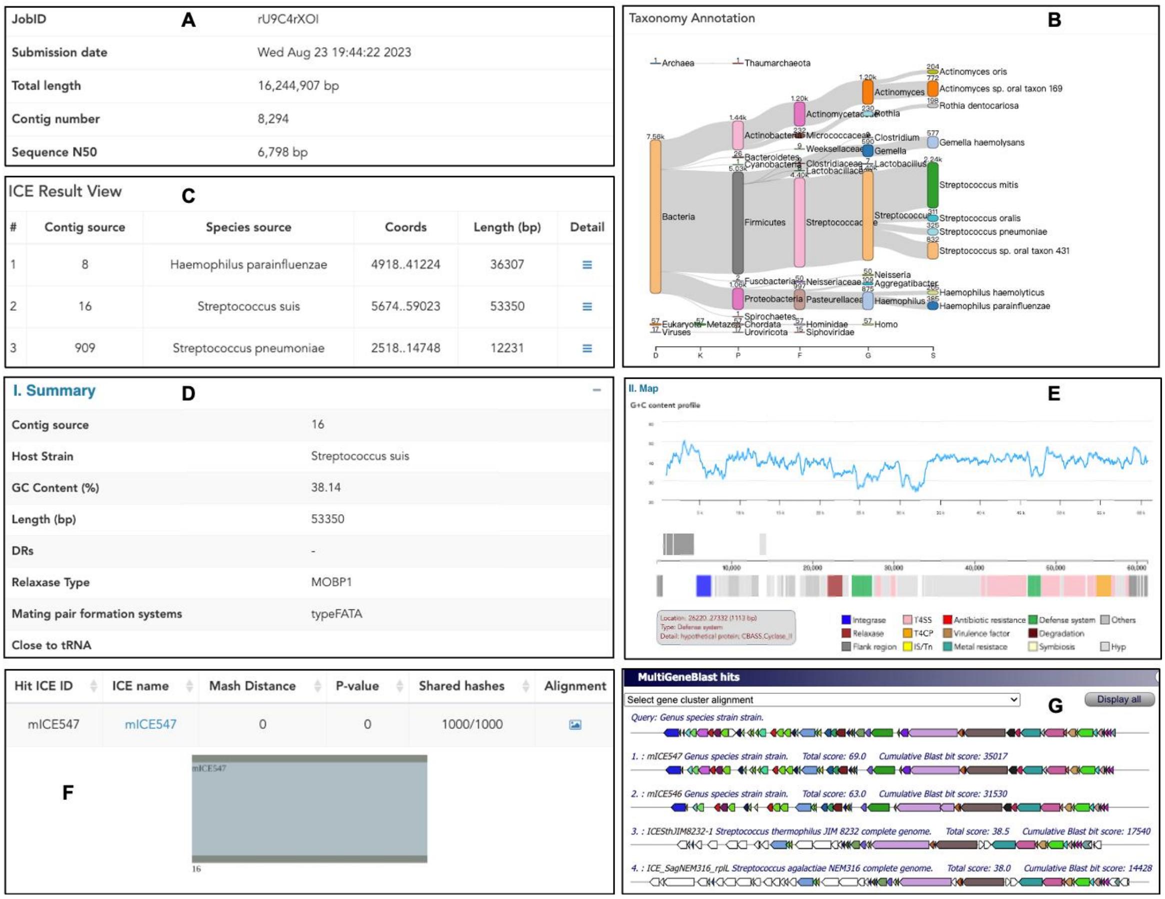

Figure S3. An overview of ICEfinder 2.0 outputs using an example of the assembled contigs from the Human Microbiome Project (HMP). The input is the oral metagenomic sequencing data (NCBI SRA: SRS146999). **(A)** The basic information of the assembled contigs; **(B)** A Sankey diagram illustrating species composition and relative abundance within the sample; **(C)** A table list of the identified ICE regions. **(D)** Detailed information about the identified ICE; **(E)** An interactive map presentation highlighting the identified ICE; **(F)** Mash-facilitated sequence comparison of the predicted ICE against the ICEberg 3.0 database; **(G)** MultiGeneBlast-facilitated sequence comparison of the predicted ICE against the ICEberg 3.0 database.

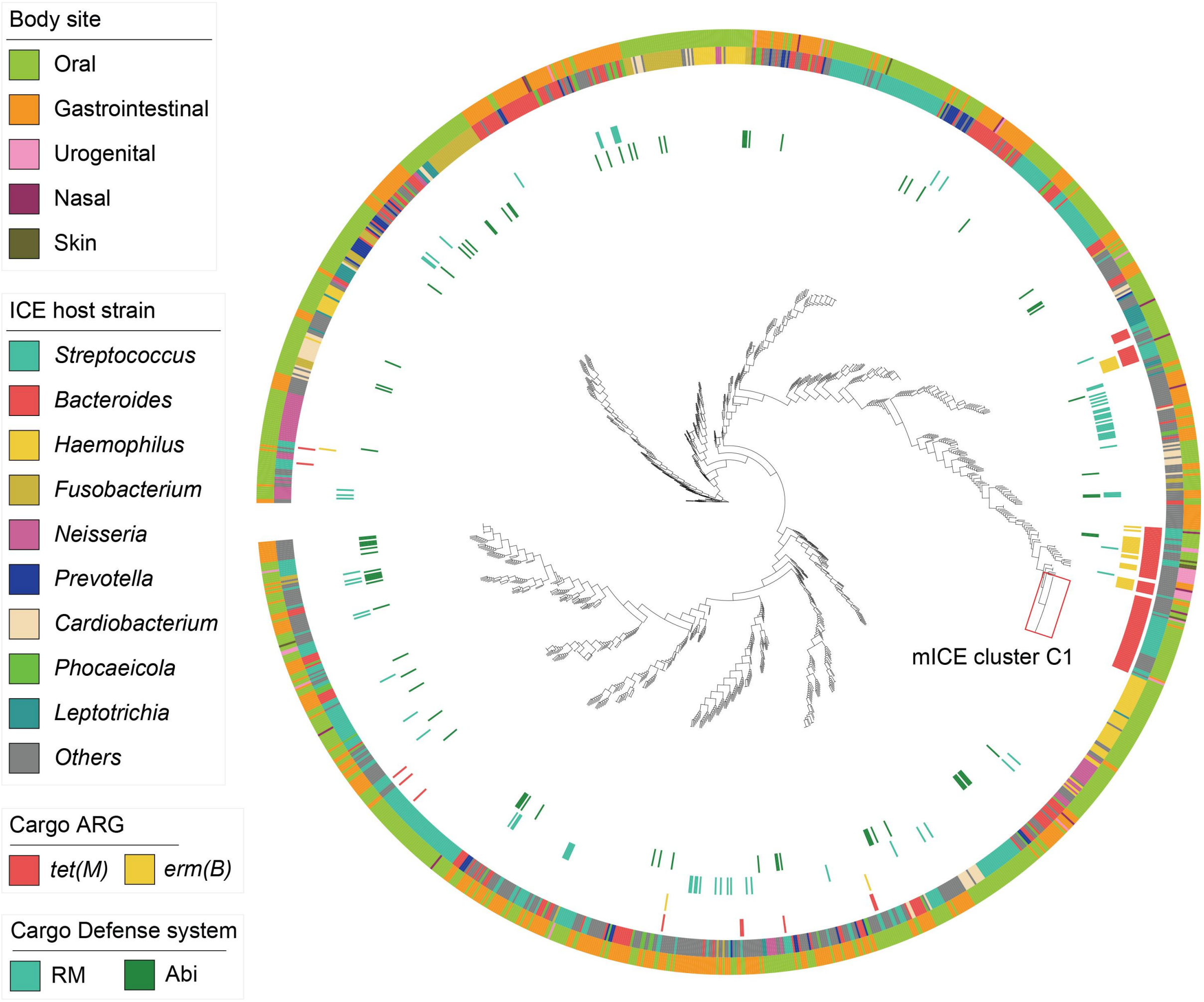

Figure S4. An overview of the 1,386 mICEs identified from the Human Microbiome Project (HMP). The phylogenetic patterns are based on the presence/absence of orthologous gene families carried by the mICEs. A binary gene presence/absence matrix was generated using OrthoMCL with default settings, and a hierarchical cluster result was constructed using iTOL. The six concentric circles from outer to inner represent body site, ICE host strain, tet(M), erm(B), Restriction-Modification (RM), and Abortive Infection (Abi), respectively. The highlighted red box signifies the largest mICE cluster, designated as cluster C1. mICEs within cluster C1 span various bacterial species and different body sites, indicating their pivotal role in facilitating the horizontal transfer of ARGs within the human microbiome.

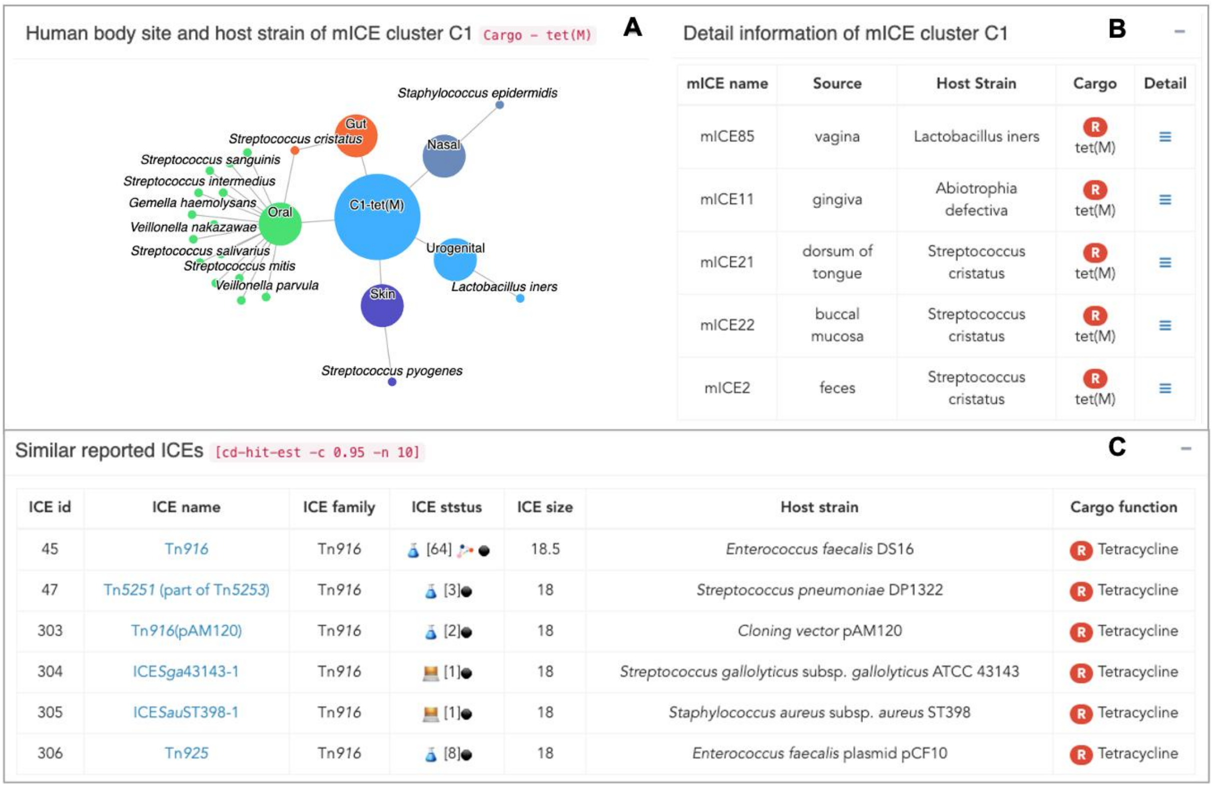

mICE name	Source	Host Strain	Cargo	Detail
mICE85	vagina	Lactobacillus iners	R tet(M)	≡
mICE11	gingiva	Abiotrophia defectiva	R tet(M)	≡
mICE21	dorsum of tongue	Streptococcus cristatus	R tet(M)	≡
mICE22	buccal mucosa	Streptococcus cristatus	R tet(M)	≡
mICE2	feces	Streptococcus cristatus	R tet(M)	≡

ICE id	ICE name	ICE family	ICE ststus	ICE size	Host strain	Cargo function
45	Tn*916*	Tn*916*	[64]	18.5	*Enterococcus faecalis* DS16	R Tetracycline
47	Tn*5251* (part of Tn*5253*)	Tn*916*	[3]	18	*Streptococcus pneumoniae* DP1322	R Tetracycline
303	Tn*916*(pAM120)	Tn*916*	[2]	18	*Cloning vector* pAM120	R Tetracycline
304	ICE*Sga*43143-1	Tn*916*	[1]	18	*Streptococcus gallolyticus* subsp. *gallolyticus* ATCC 43143	R Tetracycline
305	ICE*Sau*ST398-1	Tn*916*	[1]	18	*Staphylococcus aureus* subsp. *aureus* ST398	R Tetracycline
306	Tn*925*	Tn*916*	[8]	18	*Enterococcus faecalis* plasmid pCF10	R Tetracycline

Figure S5. Visualization of the mICE cluster C1 from the ICEberg 3.0 website. (**A**) The relationship among members of mICE cluster C1, their body site sources and their host strains, indicating a potential mobility network of those ICEs; **(B)** A tabulated list of all mICE members within mICE cluster C1; **(C)** A tabulated list featuring similarly reported ICEs in ICEberg that align with the members of mICE cluster C1.
